# Inhibition of IRAK 1/4 alleviates colitis by inhibiting TLR 4/NF-κB pathway and protecting the intestinal barrier

**DOI:** 10.17305/bjbms.2022.7348

**Published:** 2022-06-14

**Authors:** Bo Yan, Xiangjie Li, Linxiang Zhou, Yuqing Qiao, Jing Wu, Lanlan Zha, Peilu Liu, Shuai Peng, Baixin Wu, Xiaoyun Yu, Lei Shen

**Affiliations:** 1 Department of Gastroenterology, Renmin Hospital of Wuhan University, Wuhan, China; 2 Division of Gastroenterology, Wuhan Union Hospital, Tongji Medical College, Huazhong University of Science and Technology, Wuhan, China

**Keywords:** Interleukin-1 receptor-associated kinase 1/4, ulcerative colitis, TLR 4/NF-κB, intestinal barrier

## Abstract

Interleukin-1 receptor-associated kinase 1/4 (IRAK1/4) is the main kinase of the toll-like receptor (TLR)-mediated pathway, considered a new target for treating inflammatory diseases. Studies showed a significant correlation between TLRs and inflammatory responses in ulcerative colitis. Therefore, in this study, after inducing experimental colitis in mice with 3% dextran sulfate sodium (DSS), different concentrations of IRAK1/4 inhibitors were administered intraperitoneally. Then, the disease activity index was assessed, including the degree of pathological damage, by HE staining. Subsequently, while Western blotting detected the TLR4/NF-κB pathway and intestinal barrier protein expression (Zonula-1, Occludin, Claudin-1, JAM-A), real-time polymerase chain reaction detected the mRNA expression levels of IRAK1/4 and mucin1/2. Furthermore, the expression levels of Zonula-1 and occludin were detected by immunofluorescence, including the plasma FITC-dextran 4000 concentration, to evaluate intestinal barrier permeability. However, ELISA measured the expression of inflammatory factors to reflect intestinal inflammation in mice. Investigations showed that the IRAK 1/4 inhibitor significantly reduced clinical symptoms and pathological DSS-induced colitis damage in mice and then inhibited the cytoplasmic and nuclear translocation of NF-κB p65, including the phosphorylation of IκBα and reduction in downstream inflammatory factor production. Therefore, we established that the IRAK1/4 inhibitor effectively improves colitis induced by DSS, partly by inhibiting the TLR4/NF-κB pathway, reducing inflammation, and maintaining the integrity of the colonic barrier.

## INTRODUCTION

Ulcerative colitis (UC) is a chronic and non-specific inflammatory disease characterized by persistent and diffuse inflammatory changes in the colonic mucosa, usually involving the rectum and then gradually spreading throughout the colon [1]. Clinical manifestations are persistent or recurrent abdominal pain, diarrhea, mucopurulent stools, tenesmus, and systemic symptoms of varying degrees. Besides, although the incidence of UC has recently been increasing globally, its exact etiology remains unclear. In addition, UC is a process involving multiple factors, including environmental changes [2], luminal microbiota (its related antigens), and adjuvants, which activate the immune system in genetically susceptible individuals and cause immune disorders [3]. Moreover, UC treatment options currently reported include general therapy (diet-based), medications, and surgery [4]. However, because of the limitations of clinical efficacy and potential toxicity, the success of drugs is limited, causing serious medical and economic burdens to patients and the whole society.

Increasing evidence exists that the pathogenesis of UC involves changes in innate immune responses [5]. For example, Toll-like receptors (TLRs) are important components of recognition receptors and can activate innate immune responses during intrinsic immune responses. Studies specifically reported that although TLR4 expression was low in healthy human intestines and unresponsive to LPS, it was significantly increased in the intestinal tissue samples of patients with UC [6,7]. Notably, TLR4 binds non-specifically to pathogen-associated molecular patterns (PAMPs) and initiates signal transduction using the MyD88 downstream of the interleukin-1 receptor (IL-1R) for intracellular signaling. This signaling eventually leads to NF-κb activation, which triggers the release of intestinal inflammatory mediators, causing effector cells to secrete cytokines such as IL-1β, IL-6, and TNF-α. Ultimately, intestinal immune homeostasis is disrupted, leading to UC development [8].

In contrast, the interleukin-1 receptor-associated kinase (IRAK) mediates the TLRs/IL-1β pathway. Studies reported that IRAK1/4 are two important kinases downstream of the TLR pathway that demonstrate a positive regulatory effect on the signaling pathway [9,10]. Moreover, the activation of TLRs can trigger a subsequent inflammatory response, leading to disease progression [9,11]. Therefore, when PAMPS stimulates the body, TLRs activate IRAK4 by recruiting MyD88 to bind to the Toll/IL-1 receptor (TIR) domain. The activated IRAK4 further activates IRAK1 and autophosphorylates, activating NF-κB, causing upregulation of inflammatory factors and promoting inflammatory responses [12,13]. Therefore, to reduce the inflammatory response, inhibiting IRAK1/4 activity may alter the TLR/IL-1β-driven pathway. Based on these facts, this study investigated the effect of IRAK1/4 inhibitors on IRAK1/4 activity suppression in an experimental colitis model, providing a reference for UC treatment.

## MATERIALS AND METHODS

### Reagents

The IRAK1/4 inhibitor I (Cat: HY-13329, IC50: 0.3 μM (IRAK-1), 0.2 μM (IRAK-4), molecular weight: 395.41 kDa) was obtained from MedChemExpress. However, MP Biomedicals, LLC supplied the dextran sulfate sodium (DSS) (molecular weight, 36-50 kDa).

### Animals

Male C57BL/6 mice (n = 32) (6-8 weeks; 20-25 g) obtained from HFK Biotechnology Ltd (Beijing, China) were used for this experiment. Then, mice were housed in a laboratory at 21°C ± 2°C and 50% ± 5% humidity.

### Experimental design

Mice were randomly divided into four groups (n = 8) following adaptive feeding for 7 days before beginning the study.

Experimental treatments: For the control group (Con), after clean water was administered daily, 0.3 ml of 5% DMSO was injected intraperitoneally as a negative control. However, for the DSS group (DSS), after 3% DSS had been freely administered daily to induce experimental colitis, 0.3 ml of 5% DMSO was injected intraperitoneally. Then, in the IRAK1/4 inhibitor group (IRAK1/4-inh), 3% DSS was freely consumed daily, followed by the intraperitoneal administration of 0.3 ml IRAK1/4 inhibitors (10 μM [Low] and 20 μM [High]) prepared with a 5% DMSO solution. Furthermore, the dosing concentrations were based on the previous studies [14,15]. According to the above groupings and dosing patterns, treatments were administered for 7 consecutive days.

### Daily observation and sample collection

While a daily and regular observation of the appearance of mice (vitality and hair) was conducted during the experiment, each animal’s feeding and drinking conditions were recorded. In addition, body weight, fecal consistency, and rectal bleeding were monitored to assess the disease activity index (DAI) (Table 1) [16]. On the 8^th^ day, mice were executed using the cervical dislocation method, and the colorectal was immediately removed, followed by a measurement of colorectal lengths. Then, while the colon tissue was cut (1 cm) and stored in paraformaldehyde (4%) for histological examination, another part of the colon tissue was preserved at −80°C for subsequent examinations.

**TABLE 1:**
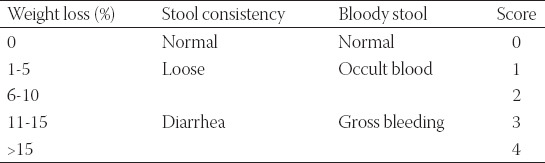
Disease activity index results

### Histopathological assessment

The tissue sample was fixed with paraformaldehyde (4%) and embedded in paraffin. Then, the tissue was cut and sectioned to a thickness of 5 mm, after which hematoxylin and eosin stained the sections. Subsequent examination of the sections was conducted under a light microscope for blind analysis. Then, histological scores are shown in Table 2 [17].

**TABLE 2:**
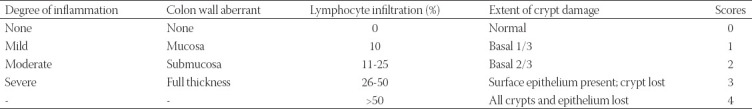
Histological colitis scores

### Myeloperoxidase (MPO) activity and inflammatory factor concentration analysis

Tissue samples were washed with preelectrodeposited phosphate-buffered saline (PBS) (0.01 M, pH = 7.4), weighed, and then cut up and ground in a full ice bath. Next, homogenization of the emulsion was conducted at 3000 rpm for 10 minutes, after which the supernatant was collected for MPO activity and inflammatory factor concentration measurements. A MPO assay kit was finally used to evaluate the MPO activity. However, inflammatory factor levels, including IL-1β, IL-6, IL-10, TNF-α, and IFN-γ, were quantified using a standard ELISA kit.

### Western blotting

Colon tissue proteins were extracted using RIPA Pierce™ Buffer (Thermo Fisher Scientific, Inc.). Then, to completely isolate cytoplasmic and nuclear proteins during the detection of NF-κB p65 nuclear translocation expression, nuclear–cytoplasmic fractionation was conducted using the NE-PER nuclear and cytoplasmic extraction reagent kit (Thermo Fisher Scientific, Cat: 78835), according to the manufacturer’s protocol. The target antibodies were rabbit polyclonal antibody, anti-(TLR4, IκBα, Zonula occludens-1; ZO-1, Occludin, Claudin-1, JAM-A), mouse monoclonal antibody, anti-MyD88, NF-κB p65, phosphorylated IκBα (*p*-IκBα), and β-actin. Finally, the relative protein expression was quantified and normalized with β-actin or LaminB (nuclear proteins) expression. All operations and antibody concentration ratio calculations were conducted according to reagent instructions.

### Quantitative real-time polymerase chain reaction (PCR)

Fresh frozen tissue stored at −80°C was used. First, the colon tissue protein-related RNA was extracted with a TRIzol reagent and transcribed with a TaKaRa reverse transcription kit. Then, PCR amplification and detection were performed using SYBR Green PCR amplification reagent on an ABI QuantStudio 6 fluorescence quantitative PCR machine, with β-actin as the internal reference. The 2−ΔΔCq method was finally used to analyze the expressions. Primer sequences used are presented in Table 3.

**TABLE 3:**
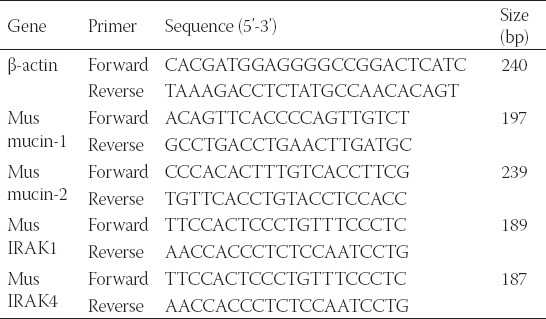
Real-time polymerase chain reaction primers

### Intestinal permeability assay

According to the previous studies, the intestinal permeability in mice was assayed with FITC-Dextran 4000 [18]. First, mice were fasted for 6 hours and then given FITC-Dextran 4000 (600 mg/kg) through intragastric administration, followed by cardiac aspiration for blood collection 1 hour later, heparinization to avoid coagulation, centrifugation, and dilution with PBS for later use. Notably, the dilution of FITC-Dextran 4000 into PBS was used to obtain a standard substance (range 50-0.312 μg/mL). Next, 100 μl of the sample, standard substance, and mouse plasma were added without intragastric administration to a 96-well plate, followed by detection using a fluorescence spectrophotometer (excitation wavelength: 485 nm and emission wavelength: 525 nm). Then, according to the standard curve obtained from the standard substance, the concentration of FITC-Dextran 4000 in each group was analyzed, and the permeability was evaluated.

### Immunofluorescence analysis

Paraffin colon tissue sections were used for ZO-1 and occludin expression immunofluorescence detection. These sections were routinely dewaxed in water and then placed on a slide rack in a pressure cooker containing citric acid antigen repair solution (pH 6.0). After capping, the pressure cooker started to air and was timed for 2 minutes. Then, the lid was opened and cooled to room temperature (23°C). The slides were later removed and rinsed with distilled water, and then, they were washed thrice for 5 minutes each with PBS. Next, the cells were incubated with an endogenous peroxidase blocking solution at a standard temperature for 10 minutes in the dark and then rinsed again. Subsequently, after drying the slides with a filter paper, two antibodies were diluted at 1:200 and 1:100, respectively, mixed evenly, dropped onto the tissues, and incubated at 4°C overnight. The next day, washing was conducted thrice with PBS, 5 minutes/time, the antirabbit and antirat fluorescent secondary antibody mixture was dropped, and incubation was continued at a standard temperature for 30 minutes. Next, washing thrice with PBS for 15 minutes was done once more and left to dry in the dark, after which fluorescent sealing tablets containing DAPI were dropped onto the slices for sealing. The slices were finally placed under a fluorescence microscope for observation and photography.

### Ethical statement

The Ethics Committee of the Renmin Hospital of Wuhan University approved all animal and experimental steps (ethical approval number: WDRM20200901).

### Statistical analysis

For data analytics and graphical representation, GraphPad Prism 8 was used. However, single-factor analyses and non-parametric tests were used for comparisons among multiple groups. Statistical significance was considered at *p* < 0.05 or *p* < 0.01.

## RESULTS

### Inhibition of IRAK1/4 alleviated the symptoms of colitis

**FIGURE 1:**
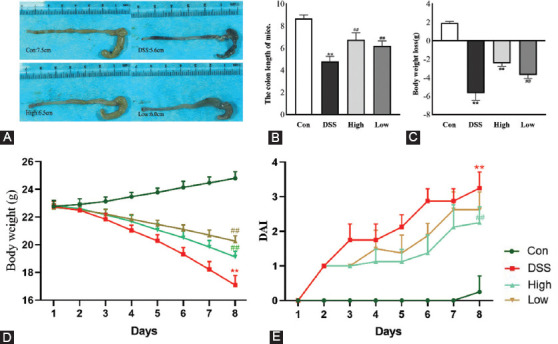
(A) Macroscopic view of the mouse colon. (B) Length of the colon. (C) Changes in the body weight of mice. (D) Weight of mice during the experiment. (E) The DAI score of mice. *p < 0.05, **p < 0.01 versus Control group, ^#^*p* < 0.05, ^##^*p* < 0.01 versus DSS group. DAI: Disease activity index.

The length of the colon is an essential index of the degree of colon inflammation. Our investigations revealed that the DSS group’s colon length was substantially shorter than that of the control (*p* < 0.01) and IRAK1/4 inhibitor (both *p* < 0.01 vs. DSS) groups, which showed significantly improved colon inflammation (Figure 1A and B). Alternatively, the mice’s body weights in each group showed different changes. We observed that while the DSS group showed a significant and continuous decrease on the 3^rd^ day of modeling (vs. Con, *p* < 0.01), similar to the IRAK1/4-inh group, the decrease degree was lower than that of the DSS group (both *p* < 0.01 vs. DSS) (Figure 1C and D). Moreover, although the DAI score was significantly increased in the DSS group (vs. Con, *p* < 0.01, Figure 1E), these symptoms were reduced in the IRAK1/4-inh group (*p* < 0.01 vs. DSS).

### Inhibition of IRAK1/4 can inhibit the TLR4-NF-κB pathway and alleviate intestinal inflammation

**FIGURE 2:**
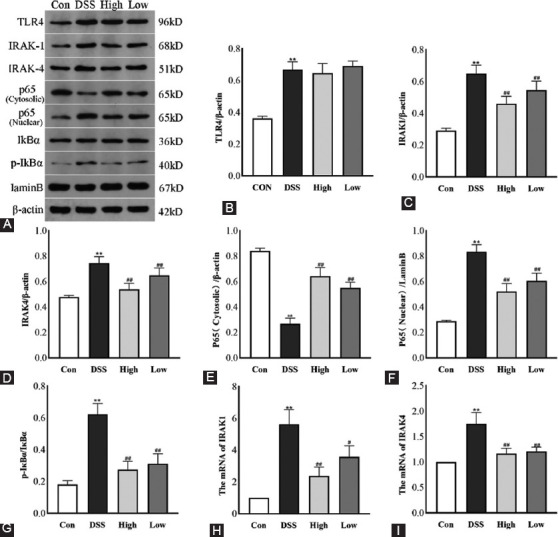
The effect of IRAK1/4-inh on the DSS-induced TLR4/NF-κB pathway in colon tissue. (A) Typical Western blotting images of TLR4, IRAK1, IRAK4, cytoplasmic p65, nuclear p65, IκBα, and p-IκBα. β-actin was the standard reference for the gray analysis. (B) TLR4, (C) IRAK1, (D) IRAK4, (E) p65 (cytoplasmic), (F) p65 (nuclear), and (G) p-IκBα/IκBα were protein relative expression levels. Also, the effect of IRAK1/4-inh on IRAK1 (H) and IRAK4 (I) mRNA levels is shown. Results are displayed as the mean ± SD (n = 3-8). *p < 0.05, **p < 0.01 versus Control group, ^#^*p* < 0.05, ^##^*p* < 0.01 versus DSS group, IRAK1/4-inh: Interleukin-1 receptor-associated kinase ¼ inhibitor.

As shown in Figure 2, the protein expression of TLR4, IRAK1/4, P-IκBα, and P65 (nuclear), including the mRNA expression of IRAK1/4 were significantly increased in the DSS group. However, the expression of IκBα and P65 (cytoplasm) was significantly decreased (*p* < 0.01, Figure 2A-F; 2H-I) in the DSS group mice compared with the control group. In contrast, although the TLR4 expression upstream of IRAK1/4 did not change significantly after treatment with the IRAK1/4 inhibitor, the expression of other up-regulated proteins decreased, including the ratio of P-IκBα/IκBα (*p* < 0.01, Figure 2G).

**FIGURE 3:**
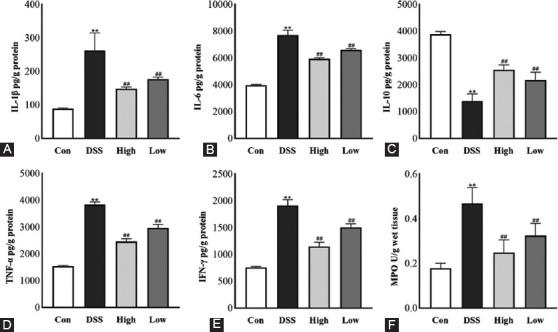
Effects of IRAK1/4-inh on inflammatory factors in mice with colitis. ELISA was used to detect the effects of IRAK1/4-inh on inflammatory factors: (A) IL-1β, (B) IL-6, (C) TNF-α, (D) IFN-γ, (E) IL-10, and (F) MPO activity. Results are displayed as the mean ± SD (n = 6-8). *p < 0.05, **p < 0.01 versus Control group, ^#^*p* < 0.05, ^##^*p* < 0.01 versus DSS group. IRAK1/4-inh: Interleukin-1 receptor-associated kinase 1/4 inhibitor.

Furthermore, results showed that although the levels of IL-1β, IL-6, TNF-α, and IFN-γ in the DSS group were significantly increased, IL-10 levels decreased (vs. Con, *p* < 0.01, Figure 3A-E). Moreover, the IRAK1/4-inh group dramatically reduced these elevated inflammatory markers and boosted IL-10 levels compared with the DSS group (*p* < 0.01). Studies reported that active UC is usually characterized by a significant neutrophil infiltrate [4,5]. Therefore, the extent of neutrophil infiltration is detected by measuring MPO activities. In this experiment, while the MPO activity of the DSS group was significantly higher than that of the control group (vs. Con, *p* < 0.01), it was dramatically reduced after the treatment with the IRAK1/4 inhibitor (vs. DSS. *p* < 0.01) (Figure 3F).

### Inhibition of IRAK1/4 can alleviate colitis mucosal damage and improve barrier function

**FIGURE 4:**
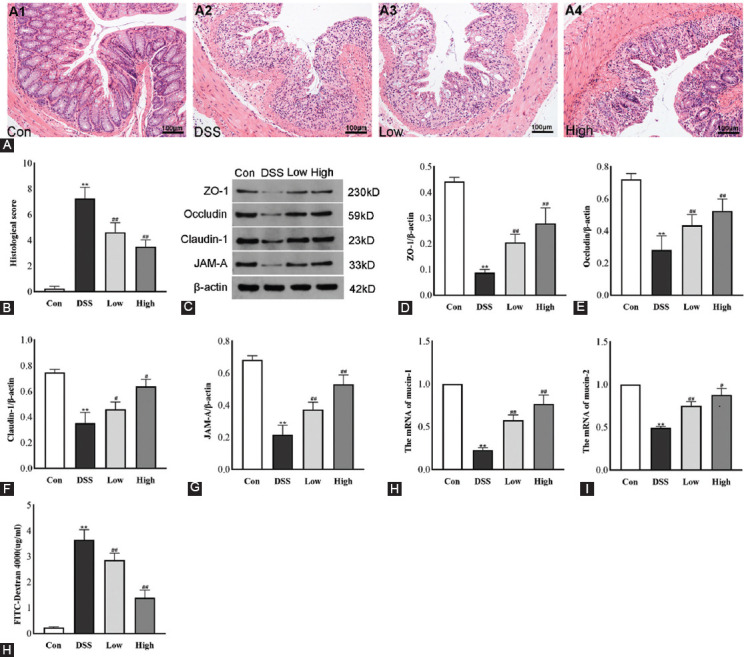
(A) HE-stain results (magnification × 200, scale bars = 100 μm). (B) Histological scores. (C) Typical Western blotting images of barrier protein and changes in (D) ZO-1, (E) Occludin, (F) Claudin-1, and (G) JAM-A relative protein expression levels. β-actin was the internal reference standard. The effect of IRAK1/4-inh on mRNA levels: (H) mucin-1, (I) mucin-2. (J) Intestinal permeability assay. Results are displayed as mean ± SD (n = 3-8). *p < 0.05, **p < 0.01 versus control group, ^#^*p* < 0.05, ^##^*p* < 0.01 versus DSS group.

As shown in Figure 4A and B, while the colonic structures of the control group were intact, the DSS group showed severe damage to the mucosal layer, including disorganized structure, complete loss of colonic glands in some mice, destruction of epithelial cells, and several inflammatory cells’ infiltration, with significantly higher microscopic histopathological scores (vs. DSS, *p* < 0.01). However, the IRAK1/4-inh group showed significantly less damage to the colonic mucosa and small intestinal villous structures than the DSS group, effectively restoring the epithelial crypt structure and reducing severe histological inflammation. Furthermore, the score was significantly reduced (*p* < 0.01).

Alternatively, as shown in Figure 4C-G, compared with the control group, while a significant inhibition of the expression of intestinal barrier-related proteins (ZO-1, Occludin, Claudin-1, and JAM-A) was observed in the DSS group (*p* < 0.01), the mRNA expression of mucin-1/2 significantly decreased (Figure 4 H-I, *p* < 0.01). However, after IRAK1/4 inhibitor intervention, compared with the DSS group, the above proteins and mRNA expression were significantly upregulated (*p* < 0.01).

In addition, the plasma concentration of FITC-Dextran 4000 was measured to evaluate intestinal permeability. Investigations revealed that the plasma concentration of FITC-Dextran 4000 in the DSS group (3.65 ± 0.37 μg/ml) was significantly higher than that in the control group (0.23 ± 0.07 μg/ml), which was by more than 15 times. However, the IRAK1/4 inhibitor group was significantly reduced (2.86 ± 0.24 μg/mL [Low] and 1.39 ± 0.28 μg/mL [High]), suppressing permeability compared with the DSS group (Figure 4J, *p* < 0.01).

**FIGURE 5:**
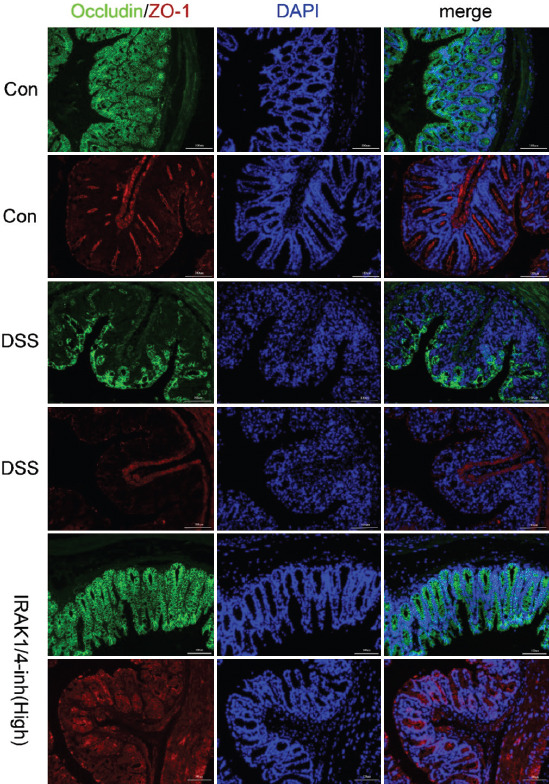
Immunofluorescence stain results (magnification × 200, scale bars = 100 μm) of the Control, DSS, and IRAK1/4-inh groups, including occludin (Alexa Fluor® 488-conjugated Goat Anti-Rabbit, green), ZO-1 (CY3 goat antirabbit, red), and the nucleus (DAPI, blue).

Subsequently, the immunofluorescence method detected the distribution of tight junction (TJs) proteins (ZO-1 and occludin) in the intestinal tissue samples of mice. As shown in Figure 5, while ZO-1 and occludin were distributed completely and densely in the intestinal mucosa of the control group (Con), the deficiency was obvious in the DSS group. Furthermore, the distribution of ZO-1 and occludin in the IRAK1/4 inhibitor group was more intact than in the DSS group.

**FIGURE 6:**
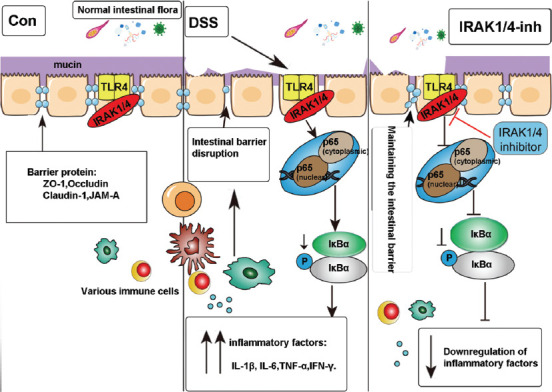
Schematic showing the possible mechanism of an IRAK1/4 inhibitor in relieving colitis. In the intestine, normal intestinal flora are found in the intestinal lumen, the outermost layer of the intestinal mucus, the central layer of intestinal epithelial cells (the most critical barrier proteins such as TJS form a barrier between cells), and the innermost layer of the lamina, which are infused with various immune cells. After DSS induction, an inflammatory response is activated through the TLR4/NF-κB pathway, destroying the intestinal barrier, suppressing pathway activation, and maintaining barrier stability. Part of this diagram refers to Li C, Ai G, Wang Y, et al. doi:10.1016/j.phrs.2019.104603.

These findings suggest that the IRAK1/4 inhibitor could reduce IRAK1/4 protein expression and inhibit the translocation of NF-κB p65 from the cytoplasm to the nucleus, including the phosphorylation of IκBα, thereby inhibiting the TLR4/NF-κB pathway and reducing inflammatory responses. Meanwhile, the inhibition of IRAK1/4 can increase the expression of TJ proteins and mucin mRNA to prevent DSS-induced intestinal integrity destruction (i.e., maintained barrier function). The possible mechanism is depicted in Figure 6.

## DISCUSSION

Although UC is a non-specific inflammatory disease whose pathogenesis has not been completely clarified, abnormal immune responses are the main causes. Studies showed that TLR4 uses downstream signaling molecules, such as MyD88 and IRAKs, to mediate the pathogen-induced activation of NF-κB in endothelial cells through the intracellular IL-1R homology domain, resulting in the generation of immune-inflammatory cytokines; thus, leading to the occurrence of UC.

Specifically, IRAKs are a class of intracellular kinases that play different regulatory roles in TIR-mediated pathways. Studies showed their close association with neoplastic diseases [19], metabolic diseases [20], and autoimmune inflammatory diseases [21]. Inhibition of IRAK1/4 activity can also alleviate inflammatory activity and reduce tissue damage. In our study, mice were successfully induced with colitis by freely administering a 3% DSS solution for 7 consecutive days through drinking and then pharmacologically inhibiting IRAK1/4 activity using the IRAK1/4 inhibitor. Next, investigations proved that inhibition of IRAK1/4 activity demonstrated a protective effect on DSS-induced colitis in mice.

Inflammatory responses are important in the development of UC. Evidence suggests [9,22] that although the TLR4/NF-κB pathway mediates intestinal inflammatory responses in patients with UC, IRAK1/4 mainly activates TLR pathway transduction. However, when pathogenic microbial ligands stimulate the organism, TLR4 activates IRAK4 by recruiting MyD88 bridging proteins to the TIR structural domain. Activated IRAK4 causes IRAK1 phosphorylation to be activated and autophosphorylated, which triggers the activation of NF-κB and other transcription factors, causing a cascade of immune-inflammatory responses. In the DSS group, our data showed that although TLR4 and IRAK1/4 were significantly elevated, IRAK1/4 expression decreased after inhibiting IRAK1/4 activity, using the IRAK1/4 inhibitor, effectively reducing the expression of NF-κB and its downstream inflammatory molecules in the intestines of colitis mice. These findings indicate that inhibiting IRAK1/4 activity alleviated inflammatory activity, which may be closely related to suppressed inflammatory responses, through TLR4/NF-κB pathway inhibition.

Next, the intact intestinal mucosa plays a key role in maintaining intestinal health. For example, it demonstrates a complex function as a semipermeable barrier that maintains nutrient absorption and immune sensing while protecting against the invasion of potentially harmful pathogenic microorganisms. Furthermore, to maintain the integrity of intestinal function and immune homeostasis, the intestinal epithelial barrier consists of a mucus layer, epithelial cells, and intercellular junctions [23]. However, the epithelium mucus layer is the first line of defense against pathogen invasion, consisting of mucins secreted by epithelial cup cells [24]. Similarly, TJ proteins, including transmembrane proteins and intracellular membrane or scaffolding proteins, have been identified as intercellular complexes at the tip of the intestinal epithelial cell junction that play a crucial roles in epithelial permeability [25,26]. Therefore, disruption of TJs can increase the colon’s permeability to harmful pathogenic microorganisms and toxins, causing an inflammatory response in the intestine and promoting UC development [27]. In turn, the impaired intestinal barrier function and increased permeability in patients with UC allow pathogenic microorganisms and toxins to cross the intestinal wall and activate TLR4, activating the NF-κB pathway, producing many inflammatory factors, and promoting the development of UC [28]. In addition, the produced inflammatory factors further worsen the disruption of epithelial barrier function, and the two, acting as positive feedback interactions, form a vicious cycle that exacerbates intestinal inflammation [28]. In this study, the mRNA expression of mucins and the intestinal TJ protein expression were significantly decreased in the DSS group. Accordingly, immunofluorescence detection showed that the fluorescence intensity and density of ZO-1 and occludin were significantly decreased. Nevertheless, after IRAK1/4 inhibitor intervention, the expression of mucin and TJs increased significantly, and the fluorescence intensity and density were enhanced significantly. This finding indicates that although the intestinal barrier function is significantly impaired when inflammation occurs in the intestine, IRAK1/4 activity inhibition can maintain the intestinal barrier’s integrity.

Picard et al. [29] observed impaired TLR-mediated secretion of inflammatory factors and chemokines in IRAK4 knockout mice and a significant reduction in the TLR-mediated secretion of proinflammatory cytokines in mice with IRAK1 gene defects. Similarly, Berglund et al. [30] showed that intestinal inflammatory cell infiltration, ulceration, and edema were reduced in IRAK1−/− mice induced by DSS. However, while Jeong et al. [31] suggested that IRAK1 promotes T cell differentiation toward IFN-γ and IL-17-producing types, Kawagoe et al. [32] showed that IRAK4 is a key enzyme in TLR signal transduction. Furthermore, they showed that the IRAK1/4-dependent pathway is involved in the early induction of genes regulating the TLR4/NF-κB pathway, promoting the release of TNF-α, IκBα, and other inflammatory factors. Based on these findings, studies concluded that the simultaneous inhibition of IRAK1/4 demonstrates better anti-inflammatory effects than either alone [33]. Researchers also reported that although pharmacological IRAK1 and IRAK4 inhibitors are less specific than knockouts, unlike IRAK4−/− mice, IRAK4-deficient patients are only susceptible to S. pyogenes and resistant to other microorganisms, including fungi, viruses, and parasites [29]. This finding may be because other junctional proteins in the human TLR signaling pathway do not signal through IRAK 4. Moreover, the susceptibility of IRAK4-deficient patients to bacteria decreases with age, approaching normal by about 14 years of age, possibly because the maturation of the adaptive immune system compensates for the immune damage caused by IRAK4 deficiency, suggesting that other immune mechanisms play an alternative role. Furthermore, the effectiveness of IRAK1/4 inhibitors has been demonstrated in autoimmune disease [31], alcoholic liver injury [34], and carotid endothelial hyperplasia [35] studies. Accordingly, in our study, although the relatively sterile environment in the laboratory may be a protective factor for experimental animals, unlike our normal living environment, mice treated with an IRAK1/4 inhibitor did not develop infections. Therefore, from the viewpoint that moderate inflammatory response itself is a protective effect on the body, pharmacological inhibition of IRAK1/4 activity may demonstrate the advantage of reducing inflammatory responses while preserving a certain degree of host defense.

In this study, although only the basic pharmacological inhibition effects of the IRAK1/4 inhibitor on IRAK1/4 activity in mice with colitis were preliminarily analyzed, the relationship between the inhibition of IRAK1/4 activity, the TLR4-NF-κB pathway activity, and improvement of intestinal barrier function was discussed. Our experiment showed that a certain concentration of IRAK1/4 inhibitor could effectively relieve DSS-induced colitis in mice. In addition, recent clinical trials of IRAK1/4-related inhibitors in RA and hematologic diseases have also been conducted [36,37], indicating the effectiveness and feasibility of IRAK1/4 inhibitors in inflammation and tumor-related diseases. However, whether these drugs are safe and effective in patients with IBD that is unclear. Therefore, further experimental studies should translate these results into clinical applications for patients with UC.

## CONCLUSION

Our study suggests that the IRAK1/4 inhibitor can reduce intestinal injury and the inflammatory response in colitis. In addition, we consider its protective effect in inhibiting the TLR4/NF-κB pathway and intestinal barrier protection function. Therefore, these findings suggest IRAK1/4 as a potential therapeutic target for UC. As a result, we consider that the IRAK 1/4 inhibitor will also be used in the future to treat UC after its efficacy is established in clinical trials of other diseases.
